# Reproducibility in pharmacometrics applied in a phase III trial of BCG-vaccination for COVID-19

**DOI:** 10.1038/s41598-023-43412-3

**Published:** 2023-09-28

**Authors:** Rob C. van Wijk, Laurynas Mockeliunas, Gerben van den Hoogen, Caryn M. Upton, Andreas H. Diacon, Ulrika S. H. Simonsson

**Affiliations:** 1 Department of Pharmaceutical Biosciences, Uppsala University, Box 591, 75124 Uppsala, Sweden; 2 TASK, Cape Town, South Africa

**Keywords:** Drug development, Biostatistics, Phase III trials, Randomized controlled trials, Infectious diseases, Respiratory tract diseases, Clinical pharmacology, Pharmacodynamics, Pharmacokinetics

## Abstract

Large clinical trials often generate complex and large datasets which need to be presented frequently throughout the trial for interim analysis or to inform a data safety monitory board (DSMB). In addition, reliable and traceability are required to ensure reproducibility in pharmacometric data analysis. A reproducible pharmacometric analysis workflow was developed during a large clinical trial involving 1000 participants over one year testing Bacillus Calmette-Guérin (BCG) (re)vaccination in coronavirus disease 2019 (COVID-19) morbidity and mortality in frontline health care workers. The workflow was designed to review data iteratively during the trial, compile frequent reports to the DSMB, and prepare for rapid pharmacometric analysis. Clinical trial datasets (n = 41) were transferred iteratively throughout the trial for review. An RMarkdown based pharmacometric processing script was written to automatically generate reports for evaluation by the DSMB. Reports were compiled, reviewed, and sent to the DSMB on average three days after the data cut-off, reflecting the trial progress in real-time. The script was also utilized to prepare for the trial pharmacometric analyses. The same source data was used to create analysis datasets in NONMEM format and to support model script development. The primary endpoint analysis was completed three days after data lock and unblinding, and the secondary endpoint analyses two weeks later. The constructive collaboration between clinical, data management, and pharmacometric teams enabled this efficient, timely, and reproducible pharmacometrics workflow.

## Introduction

Clinical trial datasets are becoming larger and increasingly complex with innovative advances in biomarker including genomic, transcriptomic, proteomic, and metabolomic measurements, mobile or wearable patient surveillance, and the use of real-world data^1,2^. The impact of larger datasets on pharmacometrics, including big data and data-mining of information sources other than clinical trial records, is expected to further increase in the coming years^3^. With more complex, non-randomized data like real-world data, transparency and reproducibility of decisions and steps in the data analysis become even more important^4^. While size and complexity of datasets and their analysis increase, the extreme scrutiny on clinical trial analysis and decision making in drug development rightfully remain unchanged. At the same time, the urgency to answer clinical questions, especially during epidemics or pandemics, increases. Thus, more complex analyses have to be performed with the same quality in a shorter time frame.

Pharmacometricians are experts in data review, processing, and analysis in clinical and preclinical pharmacology. Reproducibility, defined as reaching the same outcome when repeating an analysis^5^, is essential to all quantitative sciences, including pharmacometrics^3^. Consensus exists among the scientific community to improve reproducibility^6^. Thus, investing in developing a standardized, reproducible, and interoperable workflow will be advantageous in the long term. Essential to reproducibility is access to the data analysis workflow describing the computational steps including scripts in addition to the (raw) data^7^. Traceability and clear, transparent documentation of the workflow and the steps taken are necessary to prevent irreproducibility crises that threaten clinical and preclinical drug development^5,6,8,9^.

The coronavirus disease 2019 (COVID-19), caused by the severe acute respiratory syndrome coronavirus 2 (SARS-CoV-2), became a world-wide pandemic. It particularly put a serious strain on the health care system of South Africa^10^. Health care workers on the frontline were at high risk of contracting COVID-19^11^. Evidence of a non-specific protective effect of the anti-tuberculosis (TB) Bacillus Calmette-Guérin (BCG) vaccine against respiratory tract infections, and infections in general^12^, through epigenetic changes in the innate immune system has been reported^13–15^. In addition, epidemiology studies reported that COVID-19 burden was lower in countries with broader BCG vaccination coverage^16,17^. A double-blind, randomized, placebo-controlled trial enrolled 1,000 health care workers to investigate this hypothesis (NCT04379336, Re-BCG-CoV-19 project^18^). The primary endpoint of the trial was incidence of hospitalization due to COVID-19, with secondary endpoints including incidence of COVID-19, respiratory tract infection (RTI), and hospitalization due to all causes^19^. Participants were followed up for 52 weeks in the Western Cape, South Africa with the objective to assess in a timely manner if BCG (re)vaccination can reduce the COVID-19 burden on the health care system. To achieve this, the study started enrolment in May 2020, within two months of the first diagnosed patient in South Africa^10^, and simultaneously initiated close collaboration between the clinical, data management, and pharmacometric teams to prepare for real-time analysis and reporting.

The objective of this work was therefore to create a reproducible pharmacometric workflow for data processing, reporting, and analysis in order to support the data safety monitoring board (DSMB) and allow for efficient, timely, and reproducible data analysis. We focussed on understanding of, and confidence in handling the growing clinical trial dataset, fast and reliable frequent reporting of aggregated and per-arm data, and consistency between the reporting and the subsequent pharmacometric analysis dataset. We present here the reproducible pharmacometrics workflow that led to the rapid pharmacometrics analyses of the trial, which are not the focus on this work and the outcome of which are reported separately.

## Methods

### Workflow structure

**Figure 1:**
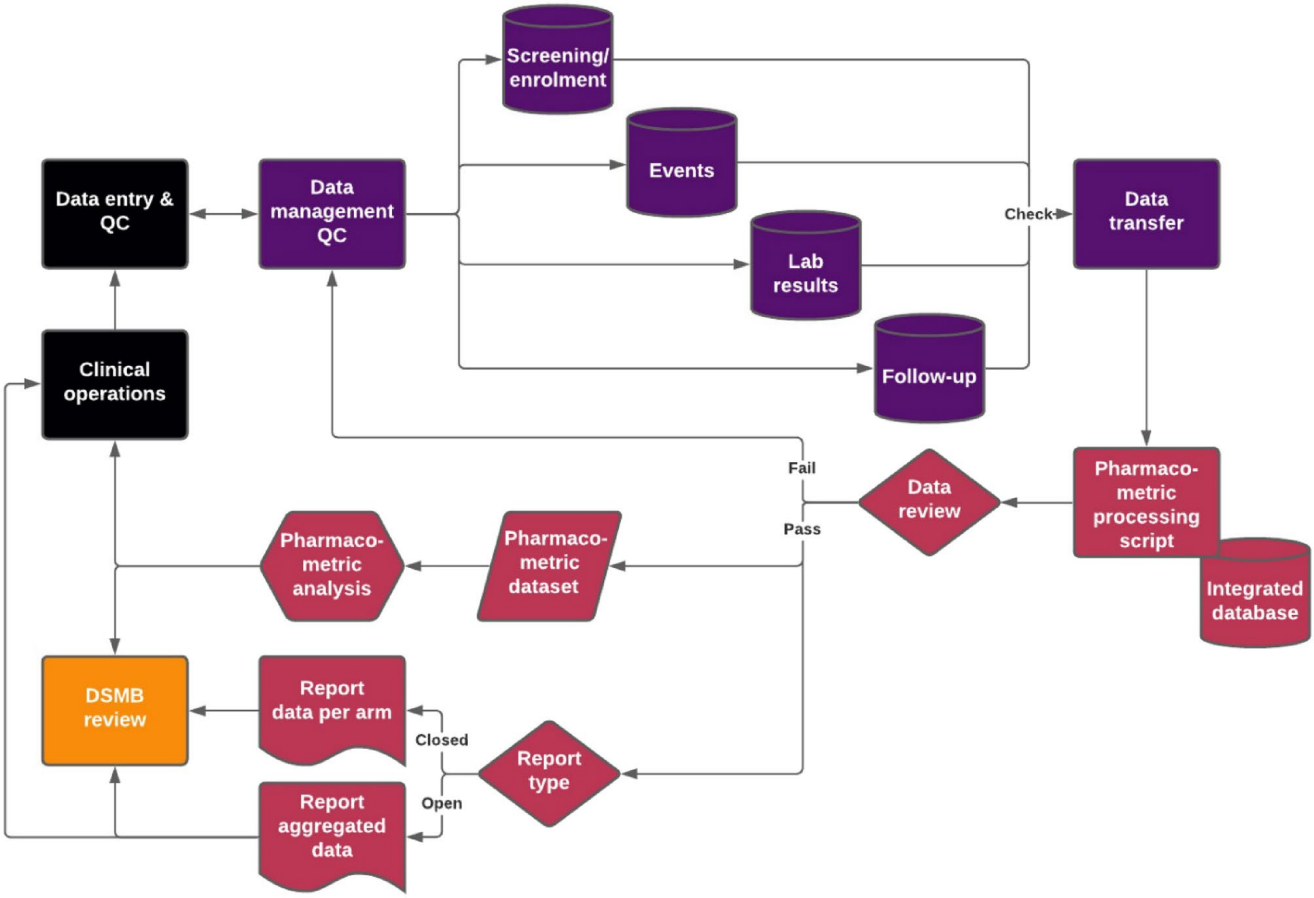
Overview of the pharmacometric reproducibility workflow. Clinical operations were the initial start of the workflow, but because of its circularity, and ongoing clinical operations throughout the trial parallel to the data processing, the workflow was performed repeatedly. The black boxes represent the clinical team, purple boxes represent the data management team, magenta boxes represent the pharmacometric team, and orange box represent the independent data safety monitoring board (DSMB). All processes were blinded until the end of the clinical trial, after which the data report per arm and the DSMB contained the unblinded data. eCRF = electronic case report form, QC = quality control.

The reproducible pharmacometric workflow was designed in consensus between the pharmacometricians, clinicians, and data managers within the project, and is shown in Fig. 1. The structure is concisely described here, with more detailed description at their respective paragraphs. Data collection from participants occurred at designated visits in the trial: screening and enrolment, monthly follow-up visits, when an event (e.g. COVID-19) occurred, and for sample collection. Data was quality controlled (QC) by the clinical and data management teams following good clinical practice (GCP) and captured in electronic case report forms (eCRFs) using an electronic data capturing tool in a virtual private cloud platform (Mobenzi Technologies (Pty) Ltd., Cape Town, South Africa). The database architecture consisted of four master databases, which were transferred to the pharmacometric team. Upon receipt of the master databases, the data was subject to the pharmacometric processing script (https://github.com/rcvanwijk/ReproducibilityPMX), which integrated the four master databases whereafter a complementary data review was performed. The pharmacometric and data management teams conferred where queries where identified, and technical inaccuracies (e.g. spelling) were corrected by the data management team in the input data, while clinical meaning ambiguity was resolved in a reproducible and annotated manner in the pharmacometric processing script.

After resolving all queries, the pharmacometric processing script produced two types of output. Firstly, the script automatically generated two data reports for frequent review; an aggregated report and a per-arm closed report. The aggregated data report, with treatment arms combined to prevent investigator unblinding or bias, was presented to the data safety monitoring board (DSMB) during the open session of their meeting, where the clinical team was present. The closed data report for the DSMB conversely showed the clinical trial data separated per arm but still blinded during the trial. Second, the pharmacometric processing script prepared for the different pharmacometric analyses, by graphical exploration of the data and data parsing to datasets for non-linear mixed effects modelling, of which the results were also presented to the clinical team and the DSMB. Collaboration on, and interoperability of, the pharmacometric processing script was essential between the different members of the pharmacometric team, which are working on the same reporting and analyses in parallel. To facilitate coding within the same script, the members of the pharmacometric team worked together in a private Github repository through Github Desktop (v.2.9.4) which at the same time ensured the script’s version control.

### Clinical trial data

The clinical trial data was captured in four master databases namely Screening/enrolment, Events, Lab results, and Follow-up, which consisted of 59 datasets in total. The Screening/enrolment database contained the participant demographics, medical and social history, and trial specific information such as inclusion/exclusion criteria, informed consent, and randomization. This database was therefore essential for covariate information to be tested in subsequent analyses. In the Events database, all information on adverse events was collected. Events were categorized as injection site reaction (ISR), respiratory tract infection (RTI), or other. Events were given a health status score by the clinical team with 0 representing healthy participant, 1 representing mild symptoms, 2 representing moderate symptoms, 3 representing severe symptoms, 4 representing hospitalization, 5 representing hospitalization with supplemental oxygen, 6 representing hospitalization with mechanical ventilation, and 7 representing death^20^. Event descriptions were standardized using the Medical Dictionary for Regulatory Activities (MedDRA, v.23.0) terminology. RTI events were followed-up and a health status score was recorded weekly. The MedDRA lower level term (LLT) was utilized to define the event type (e.g. COVID-19) while the corresponding health status score was utilized to define the event severity (e.g. 2, moderate). The Lab results database contained reports on presence of SARS-CoV-2 antibodies and latent TB infection based on interferon-gamma release assay, for which samples were taken at baseline and weeks 10, 26 and 52, or at baseline and week 52, respectively. The serology data on SARS-CoV-2 antibodies was particularly informative for asymptomatic SARS-CoV-2 infections and as a (time-varying) covariate. Lastly, in the Follow-up database, data originating from the monthly follow-up visits was recorded. This included a symptom screen and record of sick leave, as well as receipt of vaccines including influenza, BCG, or SARS-CoV-2 specific vaccinations which was important for censoring the data.

The four master databases were transferred from the data management to the pharmacometric team through secure file transfer protocol (sFTP, WinSCP v. 5.19.3^21^) and integrated into a single database based on the participant’s identifier (ID) by the pharmacometric processing script. The script was written in R (v.4.0.4^22^) using RMarkdown (v.2.7^23^) through the RStudio (v.1.4.1106^24^) interface, which allowed for clear distinction of separate code chunks per objective. The Tidyverse (v.1.3.0^25^) collection of packages including dplyr (v.1.0.5^26^), magrittr (v.2.0.1^27^), and tidyr (v.1.1.3^28^) ensured clean and transparent coding with extensive commenting for interoperability between members of the pharmacometric team. The clinical trial was performed in accordance with guidelines and regulations, and was approved by ﻿the South African Health Products Regulatory Authority (Ref: 20,200,402), Pharma-Ethics (Ref: 200,423,268) and UCT Human Research Ethics Committee (Ref: 237/2020). Informed consent was provided by all participants. Further details on the trial can be found in its primary manuscript^18^.

### Data review

The clinical team’s QC ensured all data entries to the eCRF platform corresponded to the paper source documentation. The data management’s QC subsequently checked the eCRF platform data on MedDRA coding, duplicates, incomplete or non-QC-ed records, etc. Once data had passed the clinical and data management QCs successfully, they were transferred to the pharmacometric team. The integrated database as a result of the pharmacometric processing script was subject to a data review complementary to these QCs. The pharmacometric data review had three objectives, and was performed by running the integrated data through an R-script after which results were reviewed by members of the pharmacometrics team. First, data was reviewed to comprehend the clinical meaning of the data entries, including in the context of new data and information on COVID-19 that arose throughout the trial and the ongoing pandemic. Second, once integrated by the pharmacometric processing script, the consistency between the data entries was reviewed, e.g. to not have conflicting records from different datasets for the same timepoint. Third, a check was done to ensure the eCRF data output of the eCRF platform (Mobenzi), was correctly input into the data handling software used by the pharmacometric team (R). This more technical review considered missing values, leading spaces, spelling, lower or upper cases, and numerical or character values, among others. A dedicated R chunk in the pharmacometric processing script was developed for the data review which was updated frequently. After every data transfer, the updated integrated database was reviewed using this code and queries were directed to the data management team. After all queries were resolved and data review was passed successfully, the integrated database was used for graphical and numerical exploration in the pharmacometric processing script, as well as for the DSMB report.

### Reporting

Frequent reporting on the progress of the trial was critical to identify early evidence of efficacy, if present. To that aim, the reports contained graphical and tabular exploration of the data without formal interim statistical testing. To limit the time between data transfer and distributing data reports, the pharmacometric processing script was developed to automatically generate reproducible data reports. RMarkdown was used to incorporate R-based output (numeric, graphical, tabular) with written text, which was compiled into a pdf document by knitr (v.1.33^29^). Written text and R variables were combined using the in-line R calling feature of RMarkdown. The script was coded to produce two versions of the same report based on a single switch-variable. One version of the report would show aggregated data for the trial for review by the clinical investigators, while the other version showed blinded, per arm data for review by the DSMB in their closed meeting.

### Pharmacometric analysis preparation

The pharmacometric processing script was also utilized for the pharmacometric analysis preparation. The integrated database that was input for the tables and graphs in the report, was transformed into the pharmacometric analysis datasets in NONMEM v.7.4.3^30^ format. The pharmacometric analysis datasets were used to develop a reproducible modelling workflow and strategy per endpoint (hospitalization due to COVID-19, COVID-19, RTI, hospitalization due to all causes, SARS-CoV-2 specific vaccination), including which hazard functions for time-to-event analysis to test^31^. Model scripts were drafted and tested with the interim data, and the scripts were code reviewed by a second reviewer, while the clinical trial was still ongoing, to ensure timely and efficient results upon completion of the trial. NONMEM scripts were run separate from the pharmacometric processing script. A data description table (DDT) was appended to the report for traceability of the analysis dataset variables to the source input databases. Additionally, model diagnostics using xpose4 (v.4.7.1^32^) and kernel based visual hazard comparison^33^ were included into the pharmacometric processing script.

## Results

### Transfer of clinical trial data

**Figure 2:**
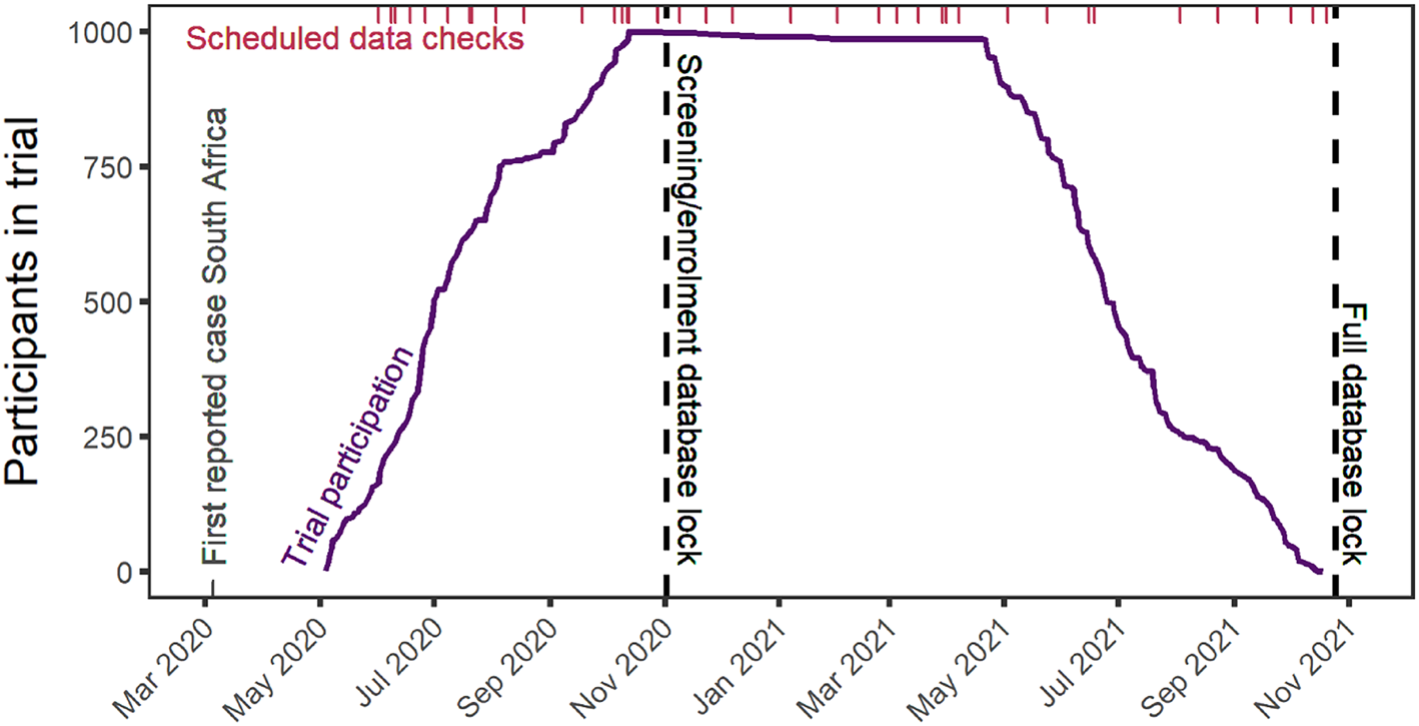
Overview of the data management throughout the clinical trial. Number of participants on trial over time is shown in purple solid line, database locks (n = 2) are shown in black dashed lines, scheduled data review (n = 41) are shown as magenta top rug plot, first reported diagnosed COVID-19 case in South Africa is shown as grey bottom axis mark for reference.

Instead of the conventional approach of receiving data for pharmacometric analysis after the trial completion, an iterative data transfer and reproducible data handling workflow was developed by consensus between the clinical, data management, and pharmacometric teams who collaborated in this clinical trial. Data QC and review responsibilities were shared between the clinical, data management, and pharmacometric teams. Figure 2 shows the participation in the trial from start of enrolment to final visit. First data was transferred once data management procedures were developed, which occurred as early as 16% enrolment. In total, 41 data transfers occurred on average every 1.8 weeks. The Screening and enrolment database was locked within three weeks after enrolment completed. Trial participation showed a slight decrease between the last participant in and first end of study visit (first participant out, April 2021) due to withdrawal and lost to follow-up or death (n = 11 and n = 2, respectively, in that interval). The full database was locked within 5 working days after trial completion, after which unblinding took place.

### Understanding and confidence in handling through data review

The frequent interim data QC by the clinical, data management, and pharmacometric teams was a time-saving investment. All records were subject to check after entry into the eCRF, and the clinical QC and data management combined found in 20.9% of the records that a correction was needed when the eCRF was compared to the paper source document. A total of 201 queries accounting for 10.7% of total records were found by the pharmacometrics team and resolved while the trial was ongoing. The last data check after the trial completed only resulted in 4 additional queries which were resolved in two days, after which the data could be locked. In addition to saving time after study completion, addressing queries while the study is still ongoing was also found to be advantageous because an incorrect measurement (e.g. weight) can still be re-measured and recorded. Pharmacometric analysis (magenta hexagon in Fig. 1) could commence practically immediately after trial completion because of this streamlined review process (other magenta, purple, and black boxes in Fig. 1). Best practices and examples of the data review are described below.

**Figure 3:**
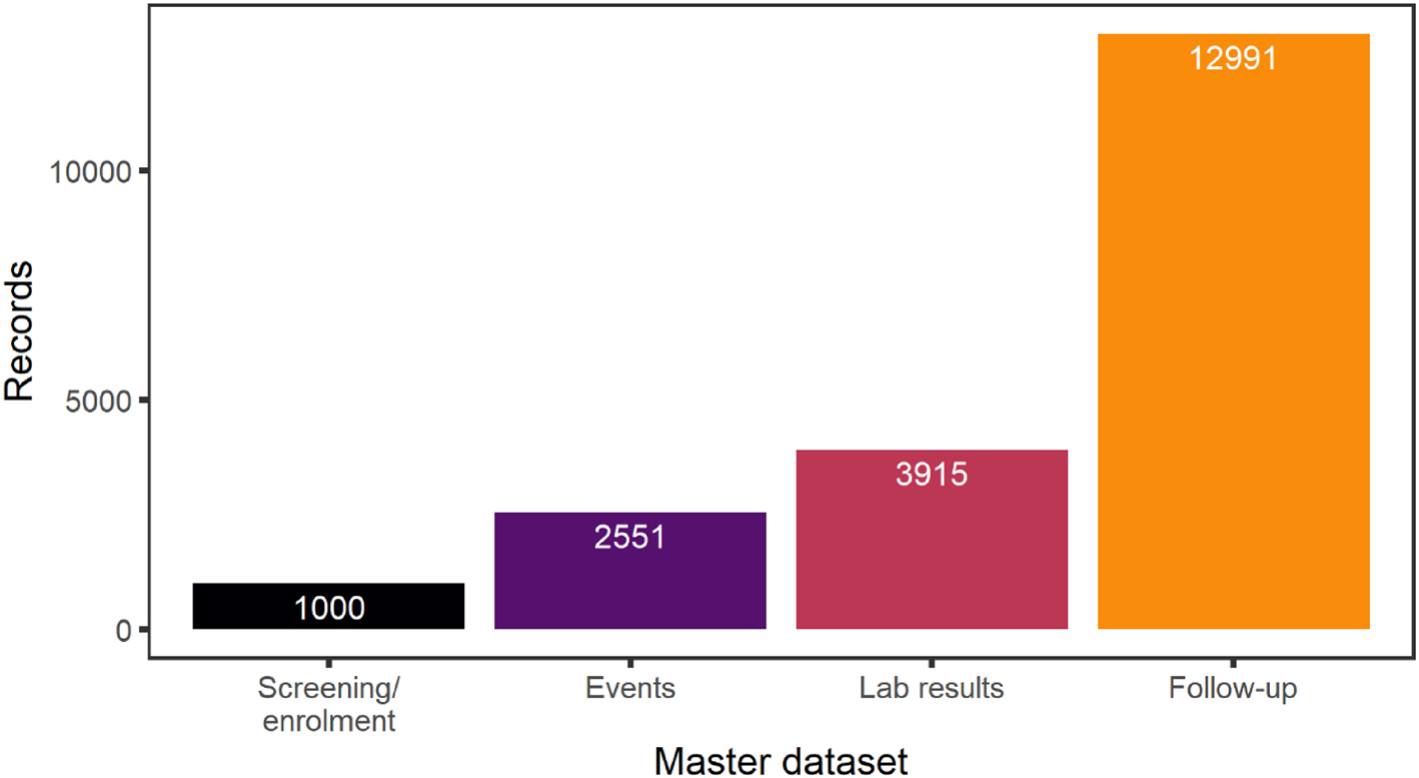
Number of eCRFs submitted per master database. Dataset architecture consisted of 4 master databases (Screening/enrolment, Events, Lab results, and Follow-up) for which the number of records is shown.

Data review was challenging because of the large size of the database. The full database consisted of 20,457 records. Figure 3 shows the database architecture including number of records per master database. The four master databases Enrolment/screening, Events, Lab results, and Follow-up, contained 24, 13, 12, and 10 datasets in .dat format, respectively (Supplementary Table I). Each dataset came with a metadata file in .stsd format reporting on each variable, possible values, and units. All records were linked through the participants ID (n = 1000) in the integrated database.

Most important in the pharmacometric data review was the understanding of the clinical meaning of the data entries. For example, COVID-19 was defined as a symptomatic disease with confirmed SARS-CoV-2 infection. As such, a COVID-19 event with a health status score of 0, or a polymerase chain reaction (PCR) confirmed asymptomatic SARS-CoV-2 infections with a health status of 1 or higher, would result in a query directed to the clinical team on how to interpret these results. The records would subsequently be corrected in the next data transfer for the health status score to reflect the event definition. Another example was post viral syndrome, i.e. long COVID. A record without a preceding COVID-19 event would also result in a query.

The consistency review between the different databases and datasets mostly focussed on the Events master database. The weekly health status score was captured in two different datasets; in the original Events dataset for the first observation(s) and thereafter in the Follow-up dataset. In the integrated datasets, these weekly health status scores were merged and checked for consistency. Where different health status scores for a single week were reported, or where the number of weekly scores did not equal the number of weeks an event was ongoing, a query was opened. Each event had a unique event number, so duplicate event numbers were flagged to the data management team. Consistency between Follow-up and Events master databases was important because participants self-reported COVID-19 events during the follow-up contact, which would result in a record in the Events master database when symptomatic. Consistency of dates between the Lab, Follow-up, and Events master databases was checked to prevent ongoing events after trial completion.

Records were checked for missing or not applicable (NA) values. Additionally, dates (negative timepoints, the same record with different dates), MedDRA event descriptions, and spelling were checked. Spelling was a noteworthy issue where COVID-19 was recorded with 63 different spelling alternatives, including COVID-19, COVID 19, COVID, COVID-19 infection, COVID-19 pneumonia, COVID-19 respiratory tract infections, while post COVID viral syndrome was recorded in 10 different alternatives. Therefore, the MedDRA term initially utilized, but unfortunately also contained two alternative spellings for both. From this insight, the MedDRA numerical codes were included into the data processing.

The initial analysis workflow evolved over time with new information and methods arising during the pandemic which were unknown at database setup. Post viral syndrome after COVID-19, also coined long COVID^34–36^, was one example, which was first reported on trial in August 2020. Discussions on long COVID developed around two points. First, the link between COVID-19 and long COVID was important to be established, by assigning those events the same event number. Second, long COVID could very well last longer than the maximum 12 weeks for which the eCRF was equipped. An additional data field was incorporated to record health status scores needed after week 12. Measurement of SARS-CoV-2 antibodies was approved by South African regulators in August 2020, and first results were reported to site in October 2020. This led to discussions around participants who were SARS-CoV-2 antibody positive at baseline, participants who were SARS-CoV-2 antibody negative after confirmed COVID-19, and on how to interpret reversal of seroconversion from positive to negative. Globally, SARS-CoV-2 specific vaccinations were first approved in December 2020 but became only available in South Africa in February 2021. Understandably, health care workers were among the first to be vaccinated with specific COVID-19 vaccines, which needed to be recorded in the database for appropriate censoring in the pharmacometric analyses. Regarding handling of events ongoing after the final (week 52) study visit consensus was reached to allow ongoing events after the final visit if the event was an important endpoint of the trial, for example COVID-19 events or respiratory tract infections in general that were symptomatic at the final visit. Acute events would be followed-up until resolution of symptoms, while chronic events like post viral syndrome would not.

### Interoperability

**Figure 4:**
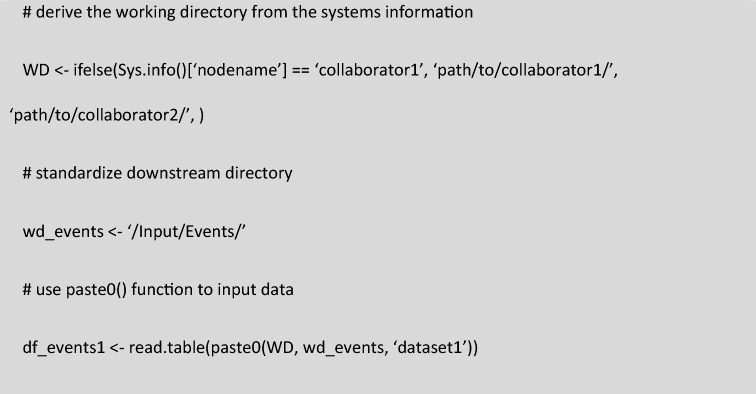
Interoperability through standardized file structure and automatic extraction of working directory using the system’s info. The ifelse() statement can be expanded with nested ifelse() statement for more collaborators.

Interoperability between members of the pharmacometric team was essential to divide the work with the short timelines. The pharmacometric processing script was stored in a private Github repository where multiple coders could work simultaneously. Through Github, changes to parts of the script by team members could be reviewed and incorporated into an updated version, all while tracking these changes and being able to revert to an earlier version in case of debugging. Additionally, the file structure between pharmacometricians was standardized, so only the path to the working directory needed to be changed relative to which all other files were inputted or outputted. The path to the working directory was automatically called at the start of the script based on an if-statement with the system’s info of the user’s machine (Fig. 4). Interoperability was also improved by using clear, transparent, and well commented coding. The Tidyverse packages including the magrittr pipe operator (% > %) allowed for better readable and interpretable code^25,27^. Interoperability between data management and pharmacometric teams was ensured by naming standards for the four master databases.

### Automatically compiled and consistent data reports

**Figure 5:**
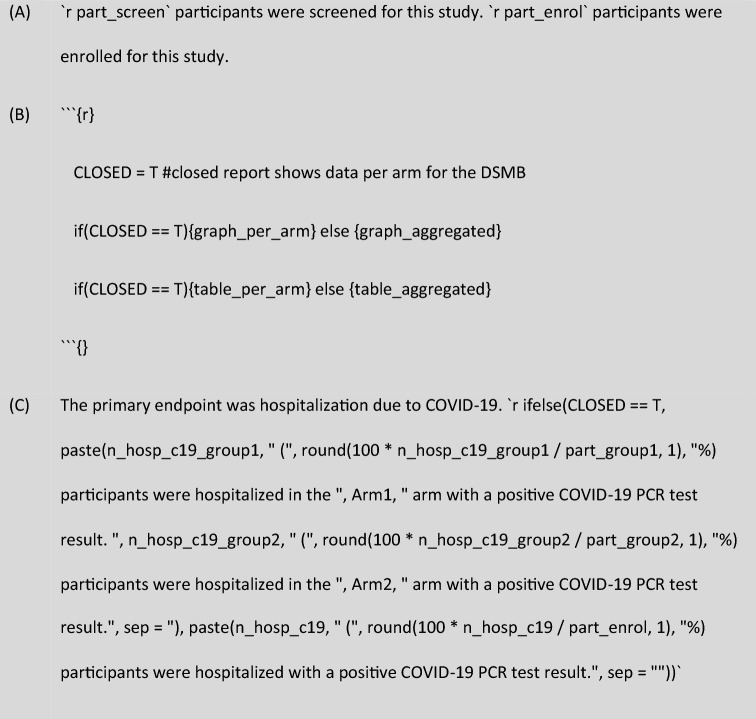
RMarkdown was used to combine text and R variables in the automatically generated report. (**A**) In-line calling of R variables to include them in a written sentence, (**B**) R variable CLOSED was used to switch between open and closed reporting using if-statements for tables and graphs called in R-chunks or (**C**) called in in-line R calls.

The pharmacometric team prepared the data reports for the DSMB to review the safety and efficacy of the ongoing trial. Because of the time-sensitive nature of the vaccination trial, initially biweekly reporting was proposed, which was later amended to a lower frequency by request of the DSMB and the clinical team because of reduced clinical urgency. Two types of reports were prepared. The open report showed the data aggregated which was open to review for the whole clinical trial, while the closed report showed the blinded data per study arm for the closed session of the DSMB. The pharmacometric processing script was developed to automatically generate a report based on the integrated database, to prevent repetitive manual report drafting with the suggested frequency. Using this method, a transparent and reproducible workflow was established from the raw eCRF input through to the DSMB report. RMarkdown was used to integrate the R-based processing of the integrated database with Markdown and LaTeX text compilers to create a report in pdf format in which the numerical, graphical, and tabular elements were automatically updated with each compilation (Fig. 5A).

To create the two versions of the report in a consistent manner, an R-variable was integrated into the relevant numerical, graphical, and tabular elements where aggregated or per-arm data was reported. This had the advantage of not having to work in two RMarkdown scripts at the same time with the risk of inconsistencies and code conflicts that occur when coding even when working as diligently as possible. As a result, the open and closed reports showed the exact same data with the only difference being the presentation of these data. The switch-variable (CLOSED) was used in if-statements throughout the report to show figures and tables either aggregated or per arm (Fig. 5B), as well as in R-code that was called in-line in the RMarkdown file (Fig. 5C).

When the DSMB meeting schedule was set, a corresponding data transfer schedule was set. On average, the DSMB received the compiled and reviewed report within 3 days after the cut-off date of the data, including the final unblinded report. The DSMB repeatedly expressed their appreciation for these “excellent turnaround times.”

### Pharmacometric analysis preparation

The pharmacometric processing script was also developed to include the pharmacometric analysis dataset creation. This resulted in a transparent, traceable, and version-controlled workflow from the raw eCRF input data to the analysis dataset in NONMEM format. Moreover, because the same script and integrated database was utilized to that aim, the datasets were consistent with the figures and tables in the DSMB reports.

The reproducible workflow and subsequent confidence in handling of the data allowed for preparation of the pharmacometric analysis of the primary and secondary endpoints while the trial was still ongoing. Based on interim graphical exploration of the data, modelling strategies were developed per endpoint including which functions to test. Model scripts were written, tested, and code reviewed before the data lock. Analysis of the primary endpoint had the highest priority. Because of the reproducible workflow and preparations for the pharmacometric analysis before the data lock, the primary endpoint analysis was completed and reviewed within three days after data lock and unblinding, and shared with the DSMB and the clinical team. Analysis of the secondary endpoints, including a total of 7 time-to-event analyses for COVID-19, RTI, and hospitalization due to all causes in both intention-to-treat and per-protocol datasets, as well as an exploratory time-to-SARS-CoV2 specific vaccination analysis, was completed and reviewed within two weeks after data lock and unblinding, and presented to the DSMB and the clinical team. As we focus here on the reproducible pharmacometrics workflow, the results of these analyses are out of scope and reported separately.

## Discussion

A reproducible pharmacometric workflow was developed here to review data iteratively during a clinical trial, report the trial data frequently to the DSMB, and prepare for the pharmacometric analyses of the primary and secondary endpoints upon trial completion to answer time-sensitive questions. Early collaboration between clinical, data management, and pharmacometric teams was established in this COVID-19 vaccination trial to ensure preparedness for the short analysis timelines. Investing in the development of this workflow paid off, as the primary endpoint analysis was completed within three days, and the secondary endpoint analyses were completed within two weeks after the data lock and unblinding. The automatically compiled open and closed reports ensured consistent and real-time presentation of the latest data when reporting to the DSMB, with on average three days between cut-off and sending the report. The DSMB repeatedly expressed their appreciation for the latest data and extensive analyses given the short timelines. The data review did not only result in confidence in handling the data, but also progressive insight with each iteration. Every single query from the data review improved the understanding of the data, their structure, and the data handling by the processing script. Additionally, through frequent interactions between the clinical, data management and pharmacometric teams, the appreciation for each other’s expertise and requirements grew which led to a very constructive environment. Drug development would benefit by moving from a conventional linear and sequential paradigm, to a more integrative approach as shown here^37^. Medical conditions that are less visible than pandemic ones might require the same urgency in answering clinical questions. Tuberculosis was the leading cause of mortality due to an infectious agent before the COVID-19 pandemic, and its burden on developing countries has only increased during the pandemic due to reduced access to care, diagnoses and treatment initiation^38,39^.

The pharmacometric reproducible workflow developed here falls within a larger context of increasing awareness in reproducibility in both data and modelling. This is especially important because of the increasing size and complexity of data acquisition and data types in drug development. The FAIR principles of findability, accessibility, interoperability and reusability of data are becoming the standard^40,41^. The Re-BCG-Cov-19 project is committed to these principles, as are progressively more publicly funded projects. For modelling, it is an encouraging first step for scientific journals to require authors to upload model scripts to reproduce the results reported in their articles^7,42^, although reproducibility to the level of successfully running these models and generating the article figures remains a concern^43^. A drug and disease model repository (DDMoRe) has been developed for pharmacometric model codes^44^, where the analysis scripts within this project will be uploaded as well, and more recently best practices in reproducibility in systems pharmacology were formulated^45^.

## Conclusion

The reproducible pharmacometric workflow we developed resulted in fast, efficient, and reliable analyses in a large clinical trial during the COVID-19 pandemic in South Africa. The constructive collaboration between clinical, data management, and pharmacometric teams enabled this efficient and robust pharmacometric data analysis which was embedded during the trial in order to support the DSMB and allowed for efficient, timely, and reproducible data analysis.

### Supplementary Information

Supplementary Information.

## Data Availability

The pharmacometric reproducibility script (without specifics of clinical data) is available at https://github.com/rcvanwijk/ReproducibilityPMX. The study data will be made available in an open access data repository upon completion of all trial analyses as per the data availability statement of the primary paper of the clinical trial^17^.

## References

[1] Danhof M, Klein K, Stolk P, Aitken M, Leufkens H (2018). The future of drug development: the paradigm shift towards systems therapeutics. Drug Discov. Today.

[2] Hulsen T (2019). From big data to precision medicine. Front Med. (Lausanne).

[3] Mentré F (2020). Pharmacometrics and Systems Pharmacology 2030. CPT Pharmacomet. Syst. Pharmacol..

[4] Rotelli MD (2015). Ethical Considerations for Increased Transparency and Reproducibility in the Retrospective Analysis of Health Care Data. Ther Innov Regul Sci.

[5] Ou YC (2013). Integration of biostatistics and pharmacometrics computing platforms for efficient and reproducible PK/PD Analysis: A case study. J. Clin. Pharmacol..

[6] Ioannidis JPA (2019). Reproducible pharmacokinetics. J. Pharmacokinet. Pharmacodyn..

[7] Stodden V (2016). Enhancing reproducibility for computational methods. Science.

[8] Wang SV (2016). Transparency and reproducibility of observational cohort studies using large healthcare databases. Clin. Pharmacol. Ther..

[9] Freedman LP, Gibson MC (2015). The impact of preclinical irreproducibility on drug development. Clin. Pharmacol. Ther..

[10] Abdool Karim SS (2020). The South African response to the pandemic. N. Engl. J. Med..

[11] Chersich MF (2020). Covid-19 in Africa: Care and protection for frontline healthcare workers. Global Health.

[12] Giamarellos-Bourboulis EJ (2020). Activate: Randomized clinical trial of BCG vaccination against Infection in the Elderly. Cell.

[13] Arts RJW (2018). BCG vaccination protects against experimental viral infection in humans through the induction of cytokines associated with trained immunity. Cell Host Microbe..

[14] Netea MG (2016). Trained immunity: a program of innate immune memory in health and disease. Science.

[15] Netea MG (2020). Trained Immunity: a Tool for Reducing Susceptibility to and the Severity of SARS-CoV-2 Infection. Cell.

[16] Miller, A. *et al.* Correlation between universal BCG vaccination policy and reduced mortality for COVID-19. *medRxiv* 2020.03.24 (2020).

[17] Escobar LE, Molina-Cruz A, Barillas-Mury C (2020). BCG vaccine protection from severe coronavirus disease. Proc. Natl. Acad. Sci. U S A.

[18] Upton CM (2022). Safety and efficacy of BCG re-vaccination in reducing COVID-19 morbidity in healthcare workers: a double-blind, randomised, controlled, phase 3 trial. EClinicalMedicine.

[19] van Wijk, R. C. *et al.* Seasonal influence on respiratory tract infection severity including COVID-19 quantified through Markov Chain modeling. *CPT Pharmacometrics Syst. Pharmacol.* **12**(9), 1250–1261. 10.1002/psp4.13006 (2023). 10.1002/psp4.13006 PMC10508522 37401774

[20] World Health Organization. WHO R&D blueprint: novel coronavirus: COVID-19 therapeutic trial synopsis. Preprint at (2020).

[21] Prikryl, M. WinSCP 2000–2022. Preprint at (2022).

[22] R statistical computing and graphics software environment. https://www.r-project.org/.

[23] Allaire, J. *et al.* rmarkdown: Dynamic Documents for R. R package version 2.1. Preprint at (2020).

[24] RStudio Team. RStudio: Integrated Development for R. Preprint at (2016).

[25] Wickham, H. tidyverse: Easily Install and Load the ‘Tidyverse’. Preprint at (2017).

[26] Wickham, H., François, R., Henry, L. & Müller, K. dplyr: A Grammar of Data Manipulation. Preprint at (2020).

[27] Bache, S. M. & Wickham, H. magrittr: A Forward-Pipe Operator for R. Preprint at (2014).

[28] Wickham, H. & Henry, L. tidyr: Tidy Messy Data. Preprint at (2020).

[29] Xie, Y. knitr: A General-Purpose Package for Dynamic Report Generation in R. Preprint at (2020).

[30] Beal, S., Sheiner, L., Boeckmann, A. & Bauer, R. J. (eds). NONMEM 7.5.0 Users Guides. (1989–2020). ICON Development Solutions, Hanover, MD, USA.

[31] Van Wijk RC, Simonsson USH (2022). Finding the right hazard function for time- to-event modeling: A tutorial and Shiny application. CPT Pharmacomet. Syst. Pharmacol..

[32] Keizer, R. J., Karlsson, M. O. & Hooker, A. Modeling and Simulation Workbench for NONMEM: Tutorial on Pirana, PsN, and Xpose. *CPT Pharmacometrics Systems Pharmacology* **2**, (2013). 10.1038/psp.2013.24 PMC3697037 23836189

[33] Goulooze SC, Välitalo PAJ, Knibbe CAJ, Krekels EHJ (2018). Kernel-based visual hazard comparison (kbVHC): a simulation-free diagnostic for parametric repeated time-to-event models. AAPS J..

[34] Mendelson M (2020). Long-COVID: An evolving problem with an extensive impact. South African Medical Journal.

[35] The Lancet Editorial (2020). Facing up to long COVID. The Lancet.

[36] Mahase E (2020). Covid-19: What do we know about ‘long covid’?. The BMJ.

[37] van der Graaf PH, Giacomini KM (2020). Clinical Pharmacology & Therapeutics 2030. Clin. Pharmacol. Ther..

[38] Geneva: World Health Organization. *Global tuberculosis report 2021*. (2021).

[39] Driessche, K. Vanden *et al.* Face masks in the post-COVID-19 era: a silver lining for the damaged tuberculosis public health response?. *Lancet Respiratory Med.* **9**, 340–342 (2021). 10.1016/S2213-2600(21)00020-5 PMC7826055 33493446

[40] Wilkinson MD (2016). Comment: The FAIR guiding principles for scientific data management and stewardship. Sci. Data.

[41] Sinaci AA (2020). From raw data to fair data: the fairification workflow for health research. Methods Inf. Med..

[42] Vicini P, Friberg LE, Graaf PH, Rostami-Hodjegan A (2013). Pharmacometrics and systems pharmacology software tutorials and use: Comments and guidelines for PSP contributions. CPT Pharmacomet. Syst. Pharmacol..

[43] Kirouac DC, Cicali B, Schmidt S (2019). Reproducibility of quantitative systems pharmacology models: current challenges and future opportunities. CPT Pharmacomet. Syst. Pharmacol..

[44] Harnisch L, Matthews I, Chard J, Karlsson MO (2013). Drug and disease model resources: A consortium to create standards and tools to enhance model-based drug development. CPT Pharmacomet. Syst. Pharmacol..

[45] Cucurull-Sanchez L (2019). Best practices to maximize the use and reuse of quantitative and systems pharmacology models: recommendations from the united kingdom quantitative and systems pharmacology network. CPT Pharmacomet. Syst. Pharmacol..

