# Supplementary material for: Reproducibility in pharmacometrics applied in a phase III trial of BCG-vaccination for COVID-19
Source: Sci Rep. 2023 Sep 28;13:16292. doi: 10.1038/s41598-023-43412-3 (PMC10539503; doi:10.1038/s41598-023-43412-3)
Supplement: Supplementary file 1 — Supplementary Information. [file 41598_2023_43412_MOESM1_ESM.pdf]

Supplementary material

Reproducibility in pharmacometrics applied in a phase III trial of BCG-vaccination for COVID-19

Rob C van Wijk[1], Laurynas Mockeliunas[1], Gerben van den Hoogen[2], Caryn M Upton[2], Andreas H Diacon[2], Ulrika S H Simonsson[1]*

*[1]Department of Pharmaceutical Biosciences, Uppsala University, Sweden, [2]TASK, Cape Town, South Africa, *corresponding author: ulrika.simonsson@farmbio.uu.se*

Supplementary table I. Full database architecture. Full database consisted of four master databases with each 10-24 datasets in .dat format.

**Master database**	**Datasets**
Screening/enrolment	copy_ICF.dat, data_entry_id.dat, demographics_contact.dat, enrolment_randomisation.dat, excl_crit.dat, exposure_assessment.dat, female_reproductive_history.dat, gen_consent.dat, Group.dat, health_questionnaire.dat, HIV_consent.dat, HIV_self_reported.dat, HIV_test_counsel.dat, incl_crit.dat, informed_consent.dat, medical_history.dat, meds.dat, participant_info.dat, qc_eCRF.dat, reimburse.dat, social_history.dat, Submissions.dat, symptom_rti.dat, vital_signs.dat
Events	data_entry_ID.dat, event.dat, event_isr.dat, event_isr_other_fu.dat, event_other.dat, event_rti.dat, event_rti_cont.dat, event_rti_fu.dat, health_status.dat, meddra.dat, participant_ID.dat, qc_eCRF.dat, Submissions.dat
Lab results	data_entry_ID.dat, participant_ID.dat, qc_eCRF.dat, serology.dat, Submissions.dat, tb_qfn.dat, visit_ID.dat

Follow-up	bcg_vaccine.dat, c19_testing.dat, data_entry_ID.dat, new_events.dat, participant_ID.dat, qc_eCRF.dat, samples.dat, Submissions.dat, symptoms_screen.dat, visit_ID.dat

bcg = bacillus Calmette-Guérin, cont = continued, crit = criteria, eCRF = electronic case report form, excl = exclusion, fu = follow-up, gen = genetics, HIV = human immunodeficiency virus, ICF = informed consent form, incl = inclusion, isr = injection site reaction, meddra = medical dictionary for regulatory activities, meds = medication, qc = quality control, qfn = quantiferon assay, rti = respiratory tract infection, tb = tuberculosis
